# Long-Term Concrete Shrinkage Influence on the Performance of Reinforced Concrete Structures

**DOI:** 10.3390/ma14020254

**Published:** 2021-01-06

**Authors:** Alinda Dey, Akshay Vijay Vastrad, Mattia Francesco Bado, Aleksandr Sokolov, Gintaris Kaklauskas

**Affiliations:** 1 Department of Reinforced Concrete Structures and Geotechnics, Vilnius Tech University (VGTU), Saulatekio al. 11, 10221 Vilnius, Lithuania; akshay-vijay.vastrad@stud.vgtu.lt (A.V.V.); mattia.francesco.bado1@upc.edu (M.F.B.); gintaris.kaklauskas@vgtu.lt (G.K.); 2 Department of Civil and Environmental Engineering, Technical University of Catalonia (UPC), c/Jordi Girona 1-3, 08034 Barcelona, Spain; 3 Laboratory of Innovative Building Structures, Vilnius Tech University (VGTU), Saulatekio al. 11, 10221 Vilnius, Lithuania; aleksandr.sokolov@vgtu.lt

**Keywords:** concrete, reinforced concrete, shrinkage, tension stiffening, concrete cracking

## Abstract

The contribution of concrete to the tensile stiffness (tension stiffening) of a reinforced concrete (RC) member is a key governing factor for structural serviceability analyses. However, among the current tension stiffening models, few consider the effect brought forth by concrete shrinkage, and none studies take account of the effect for very long-term shrinkage. The present work intends to tackle this exact issue by testing multiple RC tensile elements (with different bar diameters and reinforcement ratios) after a five-year shrinking time period. The experimental deformative and tension stiffening responses were subjected to a mathematical process of shrinkage removal aimed at assessing its effect on the former. The results showed shrinkage distinctly lowered the cracking load of the RC members and caused an apparent tension stiffening reduction. Furthermore, both of these effects were exacerbated in the members with higher reinforcement ratios. The experimental and shrinkage-free behaviors of the RC elements were finally compared to the values predicted by the CEB-fib Model Code 2010 and the Euro Code 2. Interestingly, as a consequence of the long-term shrinkage, the codes expressed a smaller relative error when compared to the shrinkage-free curves versus the experimental ones.

## 1. Introduction

### 1.1. Theoretical Background

The continuous concrete consumption growth across the globe (from buildings to bridges, from dams to power plants, etc.) has led to an increased awareness over its behavior at both earlier and later stages of its structural life-cycle. Despite such, its most common application, reinforced concrete (RC), is characterized by a complex nonlinear behavior that has still not been wholly captured. Amongst its most relevant issues, its time-dependent behavior (concrete shrinkage and creep) is still abundantly in need of investigation. This research is of crucial importance for a proper design of RC structures, as shrinkage and creep may critically affect their stress distribution when subject to a load, thus altering its deformation behavior [1,2]. Indeed, the negligence of these phenomena can potentially lead to harsh structural consequences such as member shortening, excessive deflection, and early cracking [3,4].

Generally, we refer to concrete shrinkage as the reduction of concrete volume due to the evaporation of moisture stored in its gel pores in unsaturated air environments and with no applied stress [5]. Whilst the largest amount of compressive deformations occurs in the short term (due to the withdrawal of water from capillary pores caused by the hydration of the previously unhydrated cement [6,7]), shrinkage keeps occurring along all the service life of the structure, but at a considerably reduced rate [8]. Shrinkage in concrete is influenced by several factors such as temperature, humidity, time, mix design, material characteristics, curing processes, and specimen geometry. Indeed, when dealing with larger structure sizes (skyscraper columns and long-span bridge beams amongst others), shrinkage becomes a predominant structural deformation parameter [9,10]. The observation and monitoring of this phenomenon was recently achieved with Fiber Bragg Grating (FBG) [11] and the cutting edge monitoring technology represented by distributed optical fiber sensors (DOFS) [12].

The shrinkage restraint of such a phenomenon induced by embedded elements (reinforcement bars in the case of RC structures) causes the compression of the latter and the rise of tensile stresses in the concrete surrounding it. This is aggravated by the nonuniform shrinkage occurring inside a member due to its nonuniform moisture distribution. The tensile stresses of the concrete, however, are relieved by the presence of the tensile creep phenomenon [13]. The latter can be defined as the slow and gradual deformation of the material under the continuous influence of mechanical stresses (such as the one induced by concrete shrinkage), leading, in our case, to the relaxation of the concrete. The impact of creep on shrinkage strain is dependent upon the member size, and for small sections (as the ones presented in the current article), it can be assumed null [14].

The consequences that restrained shrinkage brings to the table should be properly taken in consideration when designing an RC structure. First, among the shrinkage-restraint issues is the reduction of the tensile capacity of concrete [15] and thus the lowering of the cracking load of the structure. Secondly, if the tensile strength of concrete is to be surpassed, the development of cracks will be initiated, thus facilitating the corrosion of the reinforcement if the former is to occur in a harsh and chemically aggressive environment. This obviously would negatively influence the durability and serviceability of the structure [16,17,18]. Finally, it has been observed [19,20,21] that shrinkage negatively affects the tension stiffening potential pool of the structure.

Tension stiffening is representative of the contribution of the concrete to the stiffness and tensile strength of an RC member. Indeed, in both the elastic and postcracking phases, the concrete surrounding the rebar (the latter being situated between consecutive cracks in the second phase) relieves the latter of a certain amount of tensile stress. This phenomenon, occurring through the bond stress present between concrete and steel [22], allows an RC member to carry an additional load and its bare rebar counterpart [23,24] (thus named as tension stiffening). The latter is measured by subtracting the bare steel response from the measured member response (assuming they share the same origin).

Tension stiffening is indicated as a governing parameter for the crack resistance (in the elastic phase [25]) and deformations (later reported in [26,27]) of an RC structure. Indeed, the incorporation of the tension stiffening effect in the structural analysis of RC members makes their behavioral predictions more realistic [19] and compatible with other analyses such as the layered beam section one and the smeared finite element one [28,29].

Abundant research on the topic both in the previous century [30] and in the current one [31,32] has derived multiple tension stiffening relationships based on different assumptions and testing methodologies [33,34,35]. Worth of mention are the ones presented in the Euro Code 2 [36] (henceforth referred to as EC2) and the CEB-fib Model Code 2010 [37] (henceforth referred to as MC2010). It should be noted that the deformative response of the former has been found to be particularly stiff, especially for small reinforcement ratios [38], whereas the latter also overestimates the member stiffness only at advance loading stages [39]. However, shrinkage and creep accompany most of the abovementioned proposed tension stiffening relationships and thus were unaccounted for in their formulation, except for Bischoff [2] and Kaklauskas et al. [40] who derived shrinkage-free tension stiffening laws.

As foreshadowed earlier, neglecting the effects of shrinkage in an RC member response leads to a perceived reduction in the cracking strength of concrete and to a perceived influence of the reinforcement ratio on tension stiffening. According to Bischoff [2], these issues grow proportionally with the reinforcement ratio. Indeed, for the same amount of shrinkage, the apparent loss of tension stiffening becomes worse, as the reinforcing percentage increases (becoming unneglectable beyond 1%). Instead, as observed by other authors [35], once the shrinkage effect is removed, the tension stiffening appears to be independent from the reinforcement ratio. For all the above reasons, it is crucial to assess tension stiffening independently from the effect of shrinkage.

It should be mentioned that in most of the aforementioned experimental investigations, the shrinkage effect was studied for short-term shrinkage only (just few days). As a matter of fact, no experimental campaign studying the effect of long-term shrinkage on the tension stiffening of RC members has been reported. The present article, instead, sets itself this goal precisely.

In order to achieve it, an experimental campaign was designed and performed encompassing 14 RC tensile elements (RC ties) that cured during a time of 1947 days (5.3 years). It should be mentioned that RC ties are often used to illustrate cracking, deformation, and bond behavior of RC structures due to their simplicity and reasonably good representation of the internal distribution of forces and strains in the tensile zones of RC structures [40] (such as the one of an RC beam). After having reported the experimental load–strain curves of RC ties, the shrinkage influence on their mechanical response was mathematically extrapolated and both experimental and shrinkage-free results were finally compared with the tension stiffening predictions of the EC2 and the MC2010. This should help assess the performance of the codes when long-term concrete behavior with a magnitude of 5.3 years is concerned.

The following chapter will elucidate the well-established steps that need to be undertaken in order to remove the effect of shrinkage from tension stiffening readings.

### 1.2. Theoretical Background on the Assessment of the Shrinkage-Free Tension Stiffening Phenomenon

Considering an RC tie subjected to an external tensile load *P*, the latter is shared among the internal forces of concrete *N_c_* and steel *N_s_*_,_ as in Equation (1) (being these two defined in Equations (2) and (3), respectively):

(1) $$P=N_{c}+N_{s}$$

(2) $$N_{c}=\sigma_{ct}A_{c}$$

(3) $$N_{s}=A_{s}E_{s}\varepsilon_{s}$$

where *σ_ct_* is the average tensile stress in the concrete, *A_c_* and *A_s_* are the cross-sectional areas of concrete and steel, respectively, *E_s_* is the modulus of elasticity of steel, and *ε_s_* is the average steel strain. Thus, *σ_ct_* can be derived as per Equation (4):

(4) $$\sigma_{ct}=\frac{P-\varepsilon_{s}E_{s}A_{s}}{A_{c}}$$

The two key parameters for the derivation of tension stiffening relations are the average tensile stress in the concrete *σ_ct_* and the average member strain *ε_m_* (extracted from an RC tie test). In the past, although the predicted deformative behavior of RC structures usually ignored shrinkage strains, this has the potential to yield flawed assessments of their stress–strain behavior, crack resistance, and carrying capacity. Therefore, the present article makes the removal of the effect of shrinkage one of its priorities and the process with which this is achieved is detailed in the following.

**Figure 1:**
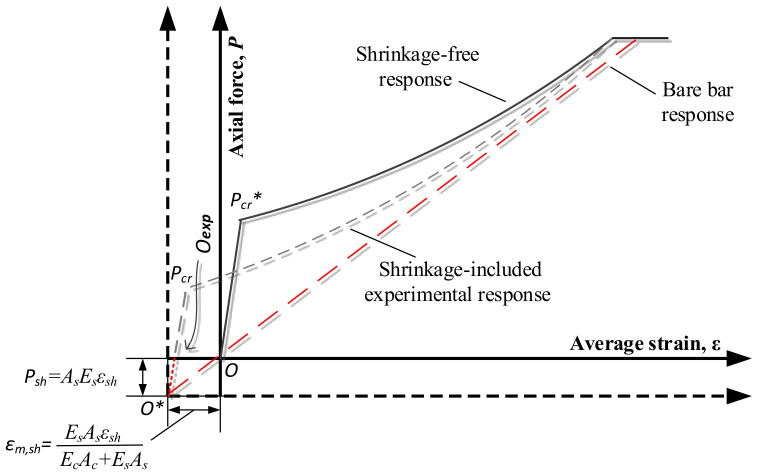
Shrinkage effect on the tension stiffening response of reinforced concrete (RC) ties.

Bischoff [2] addresses the issue with a unique method on the grounds of which the present work is based. The model has three origins (presented graphically in Figure 1):

(1) “Assumed origin O” which is the starting point of an RC tie test whenever the concrete shrinkage effects are ignored, which is how the majority of tests have been up until the present);
(2) “Experimental origin O_exp_” which identifies the starting point of the RC tie test (external load *P* = 0) except this time acknowledging the compressive effect of shrinkage on the member strains (henceforth referred to as *ε_m,sh_*) as in Figure 1;
(3) “Shrinkage-free origin O*” which identifies the true origin of the RC tie test. This time, shrinkage elimination does not apply only to the deformative response but also to the applied load. Indeed, the initial aftermath of the application of an external load *P* is simply the compensation of the abovementioned shrinkage-induced compression, henceforth referred to as $P_{sh}$ (as in Figure 1).

Here, *P_sh_* is the fictitious compressive force (defined in Equation (5)) introduced to represent the effect of the free shrinkage strain (ε*_sh_*) of the concrete on the behavior of RC members occurring in the former prior to the loading stage:

(5) $$P_{sh}=A_{s}E_{s}\varepsilon_{sh\;}$$

In this study, the free concrete shrinkage strains *ε_sh_* of the members were calculated as per the codal provisions, in particular according to the EC2 and the MC2010. On the latter two, it was observed [38] that the EC2 estimates higher free shrinkage strains than the MC2010 for higher specimen sections whilst, for smaller ones (as is the case for the present experimental campaign), the two are quite close. Furthermore, in both standards, the total shrinkage is divided into two components, namely autogenous and drying shrinkage, the second of which is particularly sensible to the environmental humidity in which the drying occurs. Since the specimens of the present study were kept in dry condition for a long time (1947 days), the amount of consequent shrinkage strain was significant.

The free shrinkage also causes an initial member shortening $\varepsilon_{m,sh}$ (expressed in Equation (6)) of which the inclusion would lead to an offset between the experimental response and the bare rebar response, as visible in Figure 1:

(6) $$\varepsilon_{m,sh}\;=\;\frac{E_{s}A_{s}\varepsilon_{sh\;}}{E_{s}A_{s}+E_{c}A_{c}}.$$

Therefore, from a graphical point of view, in order to eliminate the shrinkage effect from the tension stiffening behavior of RC elements, the origin of the load–displacement diagram can simply be shifted downwards in order to coincide with the shrinkage-free origin O*. Instead, from a mathematical point of view, in order to obtain the shrinkage free load $P^{*}$, $P_{sh}$ can simply be subtracted from the experimental tensile force applied on the member $P_{exp}$ (Equation (7)):

(7) $$P^{*}=P_{exp}-P_{sh}$$

The same procedure can be applied for the calculation of the shrinkage-free average member strain $\varepsilon_{m}{}^{*}$ as per Equation (8):

(8) $$\varepsilon_{s}{}^{*}=\varepsilon_{exp}-\varepsilon_{m,sh}$$

Figure 1 also graphically displays the shrinkage-induced apparent reduction in the cracking load of the RC member (*P_cr_* < P_cr_^*^), where *P_cr_* and P_cr_^*^ are the cracking loads of the same RC member overlooking the shrinkage effect and eliminating the calculated shrinkage from the experimentally obtained load–displacement response, respectively.

## 2. Experimental Campaign

**Figure 2:**
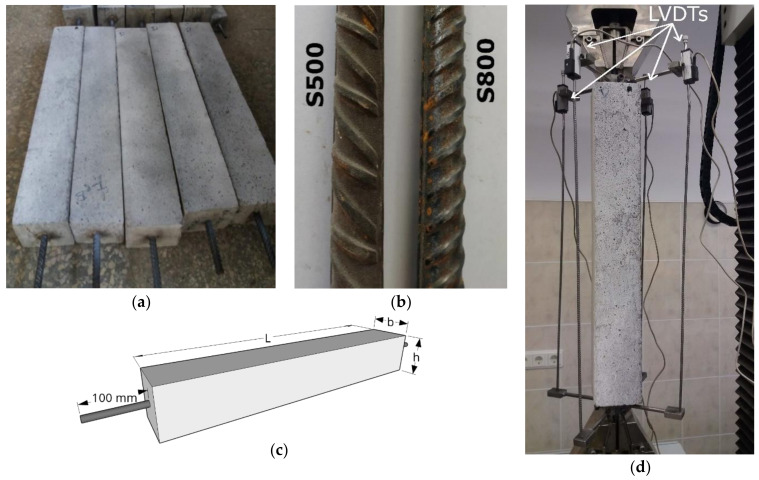
Photographs of the experimental test displaying the RC ties (**a**) and the rib pattern difference between S500 and S800 rebars (**b**). (**c**) An illustration of the RC ties to clarify their dimensional characteristics. (**d**) The RC tie clamping and testing by means of a universal testing machine (UTM).

The experimental campaign, which was the topic of the present article, consisted in the testing of 14 RC ties (visible in Figure 2a), which varied in both geometry and mechanical characteristics.

Table 1 details the concrete prism dimensions of the members (in combination with Figure 2c) and the characteristics of the embedded deformed steel rebars (diameter *Ø_s_* and resulting reinforcement ratio *ρ_s_*).

In Table 1, every member was assigned a code, where the first numerical digit was indicative of the embedded rebar diameter whilst the second represented its steel grade. The rebars were positioned longitudinally along the centroid axis of the RC tie, and in order to ensure a proper clamping during the test, their lengths were designed in such a manner that they extended beyond the concrete prism of 100 mm on both extremities (as in Figure 2c).

The members were produced with a single concrete batch using CEM II\A-LL 42.5 N cement and the PowerFlow 3100 superplasticizer. They were dried in an environment with an average temperature of 18.4 °C and an average humidity of 48.1%.

The concrete compressive strength *f_cm_* was established in accordance with BS EN 12,390 and tested on three 150 mm cubes. The modulus of elasticity *E_c_* and the tensile strength of the concrete *f_ct_* were determined according to the EC2 equation displayed as Equations (7)–(10), respectively, while Equations (9) and (10) were used for considering the time factor, as the specimens were kept for a long time (more than 28 days) before testing:

(9) $$E_{C}=22000{\left(\frac{f_{cm}}{10}\right)}^{0.3}$$

(10) fctt=βccα×fct α=23  for   t>28 days

(11) $$f_{ct}=0.3{\left[f_{cm}-8\right]}^{\left(2/3\right)}$$

(12) $$\beta_{cc}\left(t\right)=exp{\left[0.2\left(1-\frac{28}{t}\right)\right]}^{0.5}$$

The steel rebars varied both in diameter (Ø10 and Ø12) and in steel grade (S500 and S800). The contrast in the rib pattern of the different grade bars is shown in Figure 2b. The reinforcement yielding strength *f_sy_* and the modulus of elasticity *E_s_* were obtained in accordance with ISO 6892-1:2009 (specified in the standard BS EN 10025).

The specimens were subjected to uniaxial and monotonic tension loads until yielding at a rate of 0.2 mm/min by means of a universal testing machine (UTM). The average member strain was determined by means of four linear variable displacement transformers (LVDTs) fixed along the longitudinal edges of the former as represented in Figure 2d. On a side note, for future RC tie tests, an improved measurement system will be put in use. The latter would include two more LVDTs directly clamped on the rebar ends in order to avoid any measurement alteration in case of bar slippage in the gripping clamps and rigidly constraining one end whilst leaving the opposite one free and monitored by an LVDT mounted upside down in order to avoid any misalignment between the LVDT rod and the extension direction. Finally, in order to discern the location and the width of each crack appearing on the surface of the specimen, digital image correlation (DIC) monitoring was parallelly performed. Two IMAGER E-LITE 5 M cameras from LaVision (Göttingen, Germany) were fixed on a tripod stand with a distance of 0.7 m from each other and 3 m from the specimen. The cameras worked at a resolution of 2456 × 2085 pixels and at a 12.2 fps rate.

## 3. Results and Discussion

### 3.1. Standard Test Behavior of an RC Tie

**Figure 3:**
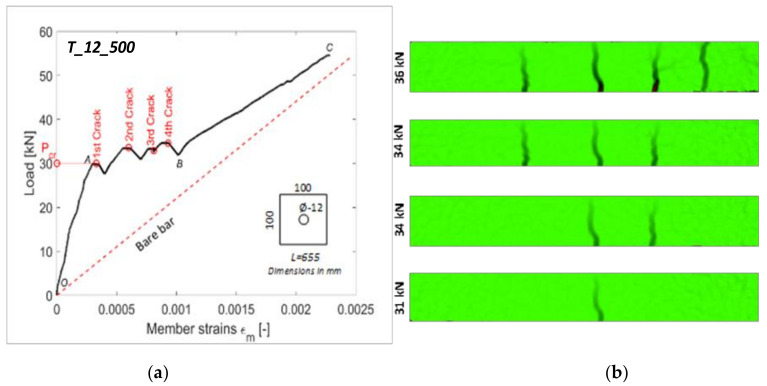
Failure behavior of a RC tie (study specimen) T_12_500: (**a**) experimental load–displacement diagram; and (**b**) development of crack with the corresponding loads through digital image correlation (DIC) pictures.

The load–average strain diagram of one of the tested members (T_12_500) is illustrated in Figure 3 in order to elucidate the cracking, deformative, and tension stiffening behavior that characterized all RC ties tests.

The first phase was defined by points O and A in Figure 3a and was defined “elastic phase” to evaluate the elastic behaviors of both constituting materials. It is at this stage that tension stiffening provided the largest contribution. Indeed, as visible in Figure 3a, the difference between the average deformation of the RC tie and the one of the bare steel rebar was largest in the interval segment OA. As described previously, the increasingly larger reinforcement stresses and strains were transferred to the concrete by means of bond stresses present on the surface between the two. This transfer continued uninterruptedly until point A, where the first crack appeared in the cross-section where the concrete stress *σ_ct_* first surpassed its tensile strength *f_ct_*. This represented the beginning of the “cracking phase” (segment AB). The corresponding load was defined as cracking load *P_cr_* which, for the study case member T_12_500, corresponded to 31 kN.

As soon as the crack appeared, the contribution of the concrete drastically decreased, thus reducing the amount of tension stiffening it provided to the member. Consequently, the average strain of the latter plummets was visible in the segment following point A. The latter occurred, whenever an RC tie is loaded with a deformation-controlled regimen, as was the case for the present test. Indeed, as soon as the UTM detected a decrease in the specimen stiffens (due to the crack formation), it suddenly decreased the applied load in order to match the defined deformation speed (0.2 mm/min in the present test). Whenever this adjustment was performed, the profile inverted its trend and increased one more. The same occurred for all the following cracks. It can be noticed that the trend of *ε_m_* progressively approached the one of the bare rebar after the appearance of every crack. This is indicative of the progressive loss of the initial extra stiffness provided by the concrete.

It was noticed for most members the first crack appeared close to the mid-section. As visible in Figure 3a, as the applied tensile stress increased in magnitude, new cracks kept appearing (their respective loads being 34, 34, and 36 kN) whilst older ones were widened. The DIC pictures of the latter with their respective loads are shown in Figure 3b. It should be kept in mind that the loads at which the cracks appeared in the DIC pictures did not necessarily correspond to the loads at which the crack-indicative profile drops appeared in Figure 3a. Indeed, the former only reported the loads at which the cracks appeared on the RC tie surface. The latter was equivalent to the drops shown in Figure 3a, only if the initial width of the crack was sufficiently wide.

Beyond point B, the lengths of the various concrete segments in which the RC tie was subdivided were insufficient for the transferred tensile stresses to reach the concrete maximum capacity, and thus, no new cracks can appeared. Indeed, in Figure 3a, no new crack-indicative drops were noticeable, and the profile acquired a linear trend. Therefore, point B represented the beginning of the third and last behavioral phase known as the “stabilized cracking stage” (section BC).

### 3.2. Test Results

The left column of Figure 3 displays all the load/average deformation graphs of the tested specimens categorized in four classes based on their reinforcement diameters and steel grades (see Table 1).

**Figure 4:**
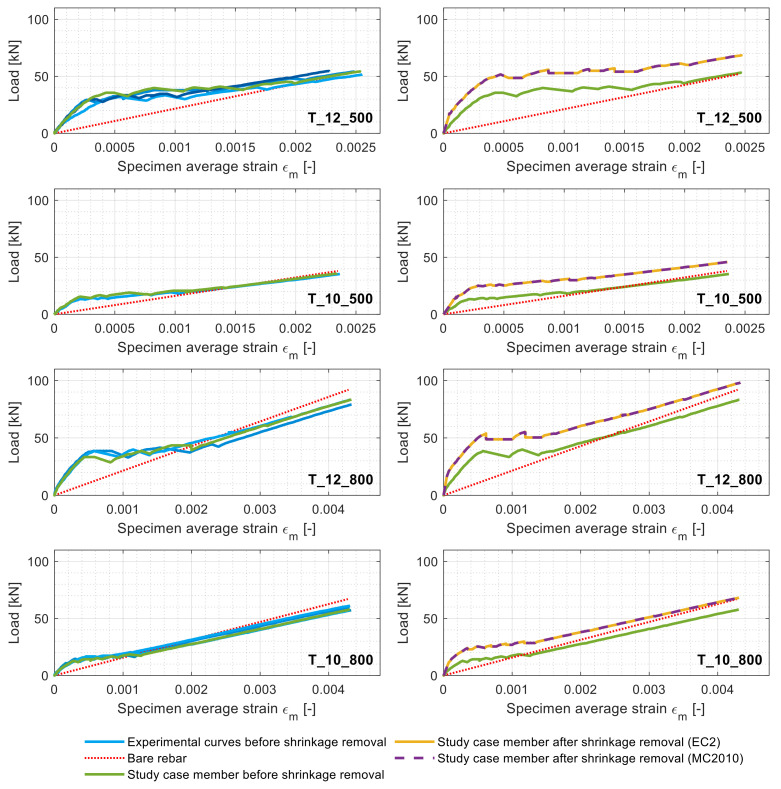
Load–specimen average strain diagrams of all the members (**left column**) and those of the study case members (**right column**) before and after shrinkage elimination.

Expectedly, a behavioral proximity can be discerned among the load–deformation graphs of the specimens pertaining to the same category. As such, for clarity purposes, only one specimen per each category (defined as “study case member” in Figure 4) was subjected to the shrinkage elimination process. The study case members were processed according to the steps detailed in Section 1.2, and the shrinkage-free profiles were reported in the right column of Figure 4 (in yellow). For the latter, the estimated *ε_sh_* after 1947 days performed with the EC2 and the MC2010 are reported in Table 2 and categorized per specimen geometry.

The process of shrinkage removal based on the EC2 and the MC2010, free shrinkage strains yielded curves that were practically identical and graphically overlapped as visible in Figure 4. Consequently, in their stead, a single shrinkage-free curve was displayed and simply named shrinkage-free experimental curve. It can be observed in Figure 4 that some of the ascending segments of the initial load–displacement curves were not perfectly linear due to the occurrence of some rebar slips in the gripping clamps. Figure 4 also shows that specimens with lower reinforcement ratios (T_12 specimens) had higher cracking loads (around 42 kN) than those with higher reinforcement ratio ones (T_10 specimens), of which *P_cr_* oscillated around 19 kN. This is due to the larger amount of concrete volume present in the former combined with the larger stiffness of its rebar. Figure 4 also displays how overlooking the shrinkage effect caused a 25% underestimation of the *P_cr_* for the T_12 specimens and about 38% for the T_10 specimens. This clearly demonstrated the significance of the long-age (5.3 years) shrinkage influence on an RC member response.

**Figure 5:**
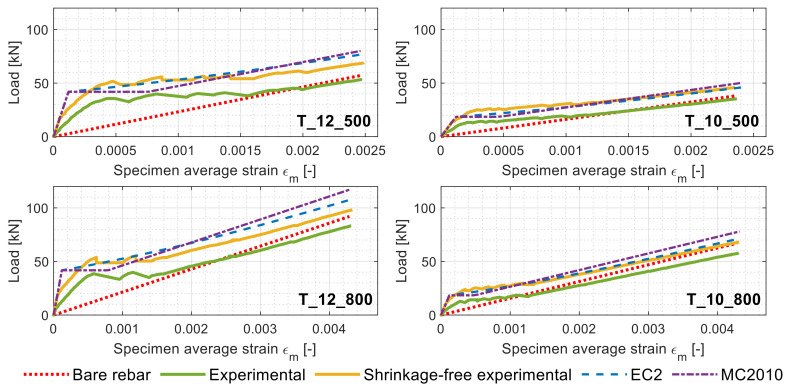
Load–specimen average strain diagrams of the study case members before and after shrinkage elimination along with the EC2 and MC2010 predictions.

Figure 5 compares the shrinkage-free load–deformation curves of the above-defined study case members against the ones predicted by the EC2 and the MC2010.

In Figure 5, it can be noticed that the experimental load–displacement curves of some members (green lines) display strains exceeding the bare rebar ones at later load levels (particularly for T_12_800 and T_10_800). This might be caused by an imperfect positioning of the LVDTs. Apart from this issue, an unexpected close match between the shrinkage-free load–displacement curves and the codal predictions (EC2 and MC2010) can be discerned for all the members, except T_12_800. The radical difference between the codal predictions and the experimental curves was highly noticeable. The tension stiffening prediction power of the codes for long-term-curing RC members is of interest, considering their equations were built on databases collecting nonshrinkage-free RC structural tests results.

### 3.3. Experiemental Tension Stiffening and Shrinkrage-Free Tension Stiffenning Relationships

**Figure 6:**
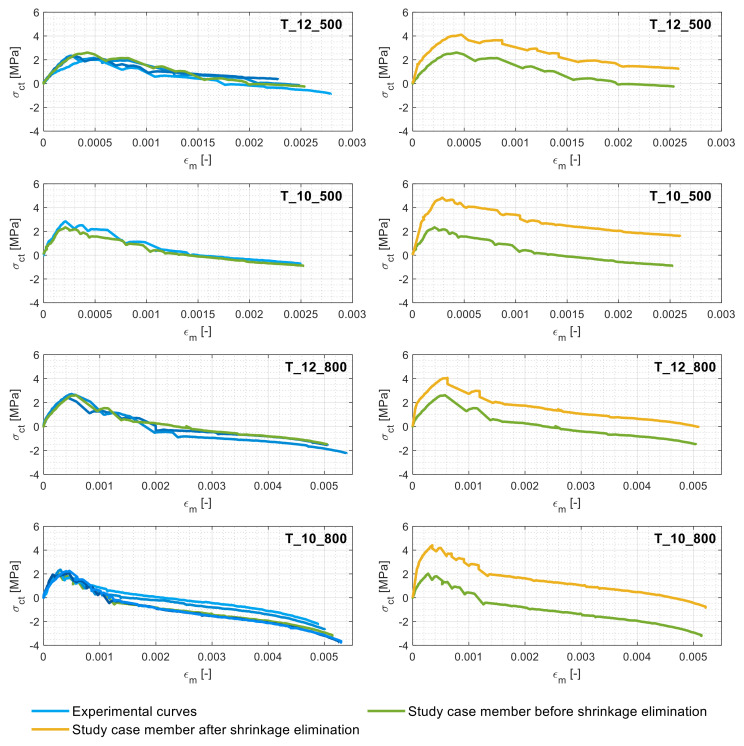
Tension stiffening behaviors of all the members (**left column**) and those of a study case member (**right column**) before and after shrinkage elimination.

The tension stiffening relationships displayed in this section were derived on the grounds of the above load–deformation curves and the Equations (3)–(12) of Section 1.2. The left column of Figure 6 shows the tension stiffening relationships of the various specimens, named as the fluctuations in the tensile stress of the concrete against the average member strain, divided in the same four categories as the ones of the previous subsection. The right column of Figure 6, instead, displays the shrinkage-free tension-stiffening curves against the experimental ones of their respective study case members.

Expectedly, the concrete gained its maximum tensile stress just prior to the appearance of the first crack (coinciding with the end of the elastic behavior), beyond which any subsequent crack led to rapid *σ_ct_* drops. Once the cracking stabilized, the tension stiffening curve does not welcome any more sudden drops but instead gradually declines due to the slow concrete/rebar bond deterioration until the reinforcement was yielded. Also expectedly, the maximum concrete stresses for all the specimens were found to be similar among all the specimens (in the range of 2–3 MPa), as they were composed of the same concrete.

**Figure 7:**
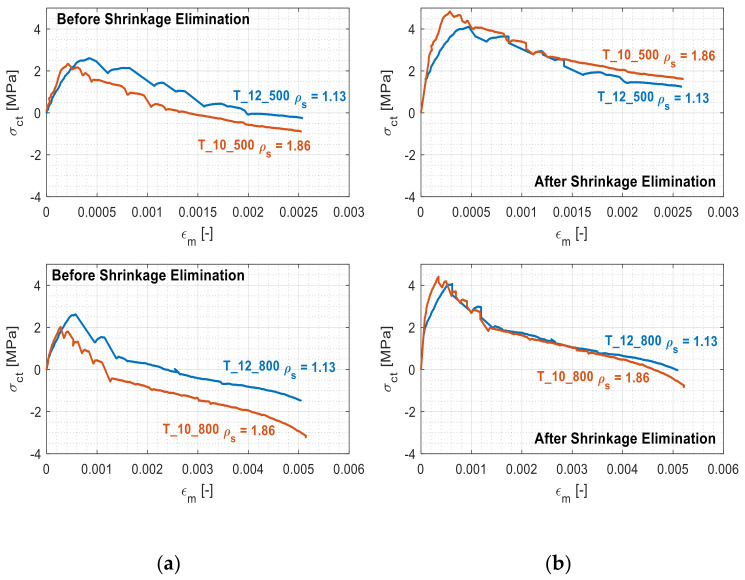
Comparison of the tension stiffening behaviors of the study case members with different reinforcement ratios before (**a**) and after (**b**) the shrinkage elimination.

The right column of Figure 6, instead, confirms how the unprocessed observations of the experimental results (thus ignoring the effect of shrinkage) led to an underestimation of the tension stiffening. In particular, it is evident that, as foreshadowed earlier, the reinforcement ratio had an undeniable influence on the tension stiffening value alteration. Figure 7 displays the tension stiffening curves of the abovementioned study case members before (Figure 7a) and after (Figure 7b) the shrinkage elimination as a function of their reinforcement ratios.

The separation present between the curves in Figure 7a is a consequence of the reinforcement ratio-induced tension stiffening alteration. Expectedly, it disappears in Figure 7b as a result of the shrinkage elimination. The apparent tension stiffening reductions were 38% and 80% for the reinforcement ratios of 1.13% and 1.86% respectively. Hence, it can be stated that for similar shrinkage-free ratios, the apparent loss in tension stiffening was more pronounced for members with a higher reinforcement ratio. Furthermore, Figure 7b, different from Figure 7a, also properly reports that the concrete still absorbed a progressively inferior amount of tensile stress *σ_ct_* until the end of the test (once again, due to the insurgence of concrete/steel slip and the consequent degradation of their bond). This is in agreement with the shrinkage-free curves of Figure 4. Indeed, the latter do not intersect the bare steel strain curve (always remaining above it) due to the continuous effect of the steel strain relaxation from the part of the concrete. Finally, the largest discrepancy between the curves in Figure 7b was concentrated in the section corresponding to their cracking phases. The reason lies in the aforementioned inferior cracking load of the T10 members, leading to a curve trend inversion in correspondence of smaller deformative values. For this reason, the T10 graphs are shifted leftwards when compared to their T12 counterparts.

**Figure 8:**
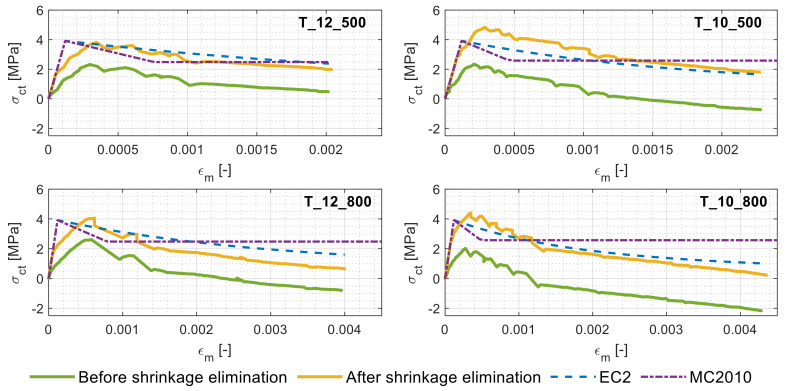
Tension stiffening behaviors of the study case members before and after shrinkage elimination along with the EC2 and MC2010 predictions.

Finally, Figure 8 plots simultaneously the tension stiffening curves extracted from the experimental campaign for the study case members, their corresponding shrinkage-free tension stiffening curves and their respective predicted tension stiffening profiles as per the EC2 and MC2010.

A substantial difference can be noticed between the predictions of the codes and the experimental response of the RC ties (green lines). With respect to the shrinkage-free response (yellow lines), instead, a much closer match can be spotted with the codal predictions. In spite of multiple research articles reporting that both the EC2 and MC2010 generally overestimate the shrinkage-free member stiffness [38,39], the “long age” could be the key explaining factor here. Further research on the topic could confirm the present hypothesis.

Figure 8 further shows that whilst the two codes yielded similar patterns in the ascending branch, they cannot be said for the descending one. Of the two, the EC2 seemed to provide closer predictions to the shrinkage-free tension stiffening curves as it includes a gradual decline in the tensile stress of the concrete in the postcracking stage (different from the MC2010).

## 4. Statistical Analysis

**Figure 9:**
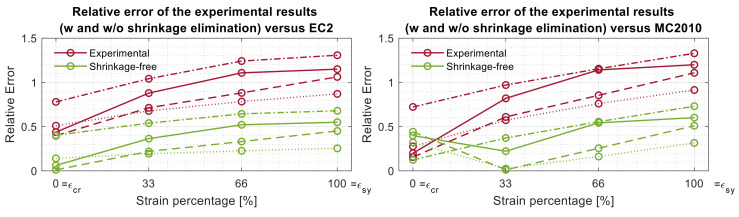
Relative errors of the experimental and shrinkage-free tension stiffening values of member T_12_500 versus the EC2 (**left**) and MC2010 (**right**) predictions.

This chapter statistically compares the experimental tension stiffening contribution of concrete *σ_ct_* with and without shrinkage elimination, against their predicted values according to the EC2 and the MC2010. Their differences were quantified through a relative error calculated as (*σ_ct,predicted_*−*σ_ct,experimental_*)/*σ_ct,predicted_*. In order to normalize the results, otherwise differing in cracking load, final load and deformation ratio, the *σ_ct,experimental_* values of the tested members were sampled at four specific test instances. The first corresponded to their cracking deformation *ε_cr_*, thus coinciding with the beginning of the descending branch of the tension stiffening graph (therefore labeled as 0% strain). The last sampling point coincided with the steel yielding strain of the members (labeled as 100%). Two intermediate levels of 33% and 66% were also introduced in order to provide an indication on the evolution of the relative errors of the readings. Figure 9 displays an example of the evolution of the relative error obtained for each of the four T_12_500 specimens along the abovementioned four stages versus the code predictions with (w) and without (w/o) shrinkage elimination.

In Figure 9, the red lines are representative of the relative errors between the experimental outputs and the relative code, whilst the green ones are representative of their shrinkage-free variances. First of all, it is observable that the relative errors of both model predictions were smaller when compared against the tested shrinkage-free results and the experimental results. As a matter of fact, the experimental results exhibited average relative errors of 0.87 and 0.79 with respect to EC2 and MC2010 predictions, respectively, and 0.35 with respect to both models for the shrinkage-free scenario. Furthermore, it is observable the relative error increased proportionally with the strain level. The relative errors of all 14 tested specimens were calculated in a similar way, and their overall mean relative error values and corresponding standard deviation for each strain stage are reported in Table 3.

**Figure 10:**
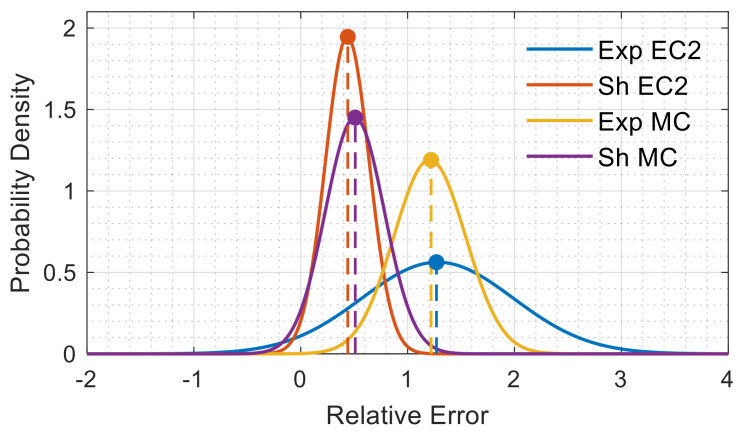
Normal distribution relative error curves between the experimental and shrinkage-free tension stiffening values versus their EC2 and MC2010 predictions.

The normal probabilistic distribution data of Table 3 are further graphically represented in Figure 10. The plots show the tension stiffening prediction errors of the EC2 and the MC2010 versus the experimental and its shrinkage-free counterpart. Furthermore, the mean relative errors are clearly indicated on the *x*-axis with the vertical dashed lines adjoining the respective peaks. It is clear that the experimental tension stiffening results had overall average relative errors of 1.25 and 1.2 with respect to the EC2 and the MC2010, respectively, whereas the shrinkage-free data were overall characterized by smaller relative errors, namely 0.45 and 0.5. Once again, it is of interest to notice the smaller prediction errors of the codes for the shrinkage-free data by considering the lack of the shrinkage elimination procedure in their constitutive equations. Additionally, a comparison of the model predictive errors concerning the raw test data elevated the MC2010 curve over the EC2 curve, as it is characterized by a smaller data spread (thus thinner bell curve) despite having a similar average.

Interestingly, the situation was reversed, whenever the elimination of shrinkage was included in Figure 10. Indeed, the EC2 exhibited a smaller data spread and a slightly higher accuracy (smaller average error) versus the MC2010.

## 5. Conclusions

The present article studied the long-age shrinkage effect on the tensile behavior, in particular on tension stiffening, of 14 tested RC tensile elements. Careful consideration of concrete shrinkage and its mechanism elimination is the key factor of this study. The assessment of the experimentally obtained results in accordance with the EC2 and the MC2010 has led to the following conclusions:

(1) The accumulated shrinkage strain during 5.3 years was quite significant and capable of making serious impact on the load–deformative behavior of the member as well as on their tension stiffening behaviors;
(2) The shrinkage effect lowered the apparent RC member cracking load. This underestimation increased with the increase in reinforcement ratio (25% for ρ = 1.13% and 38% for ρ = 1.86%);
(3) The shrinkage effect caused an apparent reduction of the tension stiffening mechanism on an average of 40% for a lower reinforcement ratio (ρ = 1.13%) and about 80% for a higher one (ρ = 1.86%);
(4) After the process of shrinkage elimination, the tension stiffening behaviors of members with different reinforcement ratios were in good agreement with each other, confirming the influence of the reinforcement ratio on the alteration of the tension stiffening effect;
(5) A statistical analysis on the tension stiffening-predicted power of the EC2 and MC2010 model codes against the experimental and shrinkage-free results showed an overall increase in relative error proportional to the strain level increase;
(6) The predictions of both codes displayed a much smaller relative error (66%) when compared against the shrinkage-free tension stiffening results than against the experimental one;
(7) According to the literature review, the previous point does not occur for short-term shrinkage, thus suggesting the increased accuracy of the model for members that include very long-term shrinkage.
(8) Among the predictions of the two models, the MC2010 one exhibited a slightly closer match to the raw test result, whereas the EC2 predictions were marginally more accurate to the shrinkage-free tension stiffening.

## Figures and Tables

**Table 1:** Geometrical and mechanical characteristics of the specimens.

Specimen Assigned Code	Number of Samples	h × b × L	*Φ_s_*	*ρ_s_*	*f_cm_*	*E_c_*	*f_sy_*	*E_s_*
		mm	mm	%	MPa	MPa	MPa	MPa
T_12_500	4	100 × 100 × 655	12	1.13	47.6	32,857	500	198,900
T_10_500	2	65 × 65 × 650	10	1.86				208,400
T_12_800	3	100 × 100 × 655	12	1.13			800	190,000
T_10_800	5	65 × 65 × 650	10	1.86				198,700

**Table 2:** Free shrinkage predicted by the Euro Code 2 (EC2) and the CEB-fib Model Code 2010 (MC2010).

Specimen Geometry	Time (days)	*ε_sh_* According to the EC2	*ε_sh_* According to the MC2010
100 × 100 × 655	1947	−0.000715	−0.000699
65 × 65 × 650	1947	−0.000722	−0.000729

**Table 3:** Mean relative error and standard deviation values for all specimens at different strain levels.

Model	Results Processing	Statistical Parameter	Strain Ratio			
			0% (ε*_cr_*)	33%	66%	100% (ε*_sy_*)
EC2	Experimental	Mean relative error (Re. Er.)	0.416	1.064	1.561	2.078
		Standard deviation (St. Dev.)	0.156	0.295	0.586	0.976
	Shrinkage-free	Mean Re. Er.	0.231	0.324	0.488	0.698
		St. Dev.	0.112	0.199	0.336	0.493
MC2010	Experimental	Mean Re. Er.	0.470	0.944	1.294	1.644
		St. Dev.	0.209	0.250	0.313	0.445
	Shrinkage-free	Mean Re. Er.	0.331	0.275	0.533	0.884
		St. Dev.	0.211	0.189	0.289	0.376

## Data Availability

The data presented in this study are available on request from the corresponding author.
